# Clinical tooth segmentation based on local enhancement

**DOI:** 10.3389/fmolb.2022.932348

**Published:** 2022-10-11

**Authors:** Jipeng Wu, Ming Zhang, Delong Yang, Feng Wei, Naian Xiao, Lei Shi, Huifeng Liu, Peng Shang

**Affiliations:** ^1^ Shenzhen Institutes of Advanced Technology, Chinese Academy of Sciences, Shenzhen, China; ^2^ Department of Pediatrics, Zhongshan Hospital Xiamen University, Xiamen, China; ^3^ Department of Burn Surgery, The First People’s Hospital of Foshan, Foshan, China; ^4^ Department of Neurology, The First Affiliated Hospital of Xiamen University, Xiamen, China; ^5^ Dental Medicine Center, The Second Clinical Medical College of Jinan University, Shenzhen People’s Hosipital, Shenzhen, China

**Keywords:** tooth segmentation, decoder, local enhancement, ASPP, CNN

## Abstract

The tooth arrangements of human beings are challenging to accurately observe when relying on dentists’ naked eyes, especially for dental caries in children, which is difficult to detect. Cone-beam computer tomography (CBCT) is used as an auxiliary method to measure patients’ teeth, including children. However, subjective and irreproducible manual measurements are required during this process, which wastes much time and energy for the dentists. Therefore, a fast and accurate tooth segmentation algorithm that can replace repeated calculations and annotations in manual segmentation has tremendous clinical significance. This study proposes a local contextual enhancement model for clinical dental CBCT images. The local enhancement model, which is more suitable for dental CBCT images, is proposed based on the analysis of the existing contextual models. Then, the local enhancement model is fused into an encoder–decoder framework for dental CBCT images. At last, extensive experiments are conducted to validate our method.

## 1 Introduction

In recent years, application of computer tomography (CT) Gao et al. (2021) has become increasingly perfect, which can provide three-dimensional images for partial magnification observation, and its clinical penetration rate is getting higher and higher. However, due to further breakthroughs in image reconstruction technology, metal and bone artifacts have always been restricted to their full use. The emergence of CBCT Schulze et al. (2011) has partially solved the aforementioned problems. Dental CBCT uses 3D cone beam X-ray scanning instead of the 2D fan beam scanning of traditional CT. The significant difference is that the projection data of tomographic CT are one-dimensional, and the reconstructed image data are two-dimensional. Due to the accumulation of successive two-dimensional slice forms, the reconstructed three-dimensional image and the image metal artifacts are relatively heavy, while the projection data of CBCT are two-dimensional, and the three-dimensional image is directly obtained after reconstruction, which greatly solves the problem of artifacts. In addition, the thickness of CBCT layers can be as low as 0.1 mm, which provides better imaging quality for complicated tissue structures such as teeth or jaws.

Digital dental care provides assisted patient care and has become a reality based on computer vision science. As a newly developed medical imaging technology, CBCT reconstructs the patient’s anatomical structure through X-rays, enabling stomatologists to observe the arrangement of tooth roots. On this basis, the stomatologist can measure the patient’s teeth, including the shape, position, and main axis direction of the teeth Wexler et al. (2020). For children, the tooth arrangement is particularly difficult to observe. Children undergo growth and tooth replacement, with a high incidence age for dental caries, enamel hypoplasia, early eruption, dental trauma, and occlusal disorders. Therefore, dental imaging examinations are unavoidable and more frequent in children than in adults, resulting in more significant long-term harm. At the same time, children are more sensitive to radiation, their age and body size are smaller, and the exposure per unit of the body surface area is more significant under the same radiation dose. In addition, children are less likely to cooperate and cannot tolerate prolonged examinations. CBCT under semantic segmentation does not require children to pose particular poses, leading to a shorter examination time than that of traditional methods, which can complete the analysis better.

The tooth CBCT image segmentation can establish a three-dimensional model of the tooth, and then the dentist can quickly and accurately measure the patient’s teeth in all directions (Park and Kwak, 2019). If dentists are required to manually segment teeth in CBCT images, it is expensive and impossible to be clinically acceptable. CBCT images are three-dimensional images with a sub-millimeter resolution, and a CBCT image usually contains 28–32 teeth (Gao and Chae, 2010). It usually takes a professional dentist several days to perform high-precision segmentation of each tooth in a CBCT image. Therefore, automatic or semi-automatic tooth segmentation algorithms can replace many repeated calculations and annotations in the manual segmentation process by doctors and quickly achieve high-precision tooth segmentation in CBCT images, which is of great clinical significance.

Researchers have applied many methods to segment teeth in the past few decades, including threshold-based (Ramesh et al., 1995), edge-based (Lin et al., 2012), region-based (Akhoondali et al., 2009), and cluster-based (Alsmadi, 2018) segmentation methods. However, those classical algorithms exhibit limitations when dealing with the aforementioned challenging conditions, e.g., varying intensities, unclear boundaries, and the presence of metal artifacts. Moreover, classical algorithms often require manual seed points to perform tooth segmentation, making these methods incapable of achieving fully automated segmentation. More recently, some literature based on the deep convolutional neural network (DCNN) (Krizhevsky et al., 2012) was proposed, resolving individual tooth segmentation. With the continuous deepening of the DCNN, tooth segmentation has rapidly developed. In particular, the fully convolutional network (FCN) (Long et al., 2015) provides a new research idea for tooth segmentation. In particular, FCN replaces the fully connected layer with a fully convolutional layer to realize the conversion of the input image to the output image. Meanwhile, DCNN-based methods (Ronneberger et al., 2015); Yu et al., 2020); Ma et al., 2021) can train the segmentation model using pixel-level annotation information as labels, including category, spatial information, and location information. Owing to richer object prior information, DCNN-based methods can adapt to semantic parsing of complex scenes. DCNN-based methods mainly have the following advantages: 1) depth features have strong characterization ability. A deep learning dense connection network can realize automatic extraction of convolutional features in CBCT images. 2) The image has local invariance. Deep learning technology is developed from natural image analysis, and local invariant features of realistic images are also applicable to dental CBCT images. 3) The network has flexibility. The framework can flexibly apply to tooth CBCT image segmentation according to different task requirements.

Based on the robust feature extraction of deep learning networks, many researchers (Im et al., 2022); Hao et al., 2022) focused on how to improve tooth segmentation. Xu et al. (2018) proposed a boundary-aware method to improve the efficiency of feature extraction. Nguyen et al. (2020) segmented the alveolar bone and located the alveolar crest *via* a convolutional neural network. Cui et al. (2019) extracted the edge map from the feature layers to enhance image contrast along shape boundaries. Zhao et al. (2020) used the global and local attention modules to locate the tooth region. Cui et al. (2021a) exploited comprehensive semantic information of tooth segmentation by a generative adversarial network. Cui et al. (2021b) designed a tooth centroid voting scheme for the detected tooth and then used a confidence-aware cascade segmentation module to segment each tooth. Although researchers have made considerable efforts to enhance the accuracy of tooth segmentation, automatically and robustly extracting a tooth from dental models remains a challenging task. First, some patients have complex dental conditions, e.g., crowded, missing, and misplaced teeth. Meanwhile, adjacent teeth are often irregular and difficult to separate. Second, the teeth and gums are short of noticeable shape changes at the boundary and the distinction of geometric features, making it difficult for the boundary-based segmentation method to distinguish these two parts.

**FIGURE 1:**
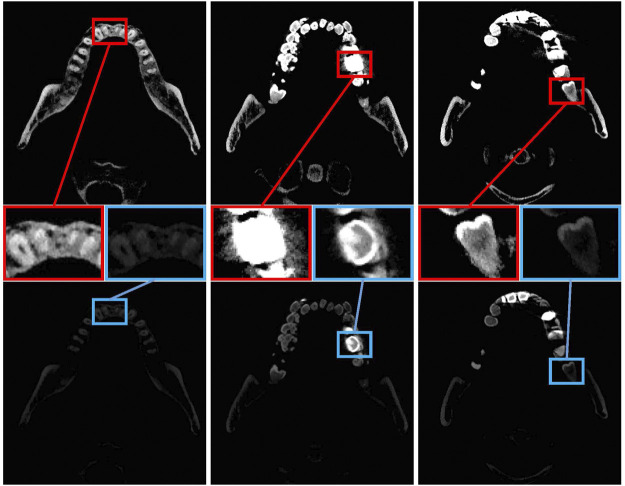
Tooth CT images of three slices. The top and bottom lines represent the CBCT images of teeth obtained by setting different window widths and window positions. Teeth or metal may not be separated when the window width is not set correctly. The top line set the window width and window level to 3,010 and 2,006. In the bottom line, the window width and window level are set to 3,074 and 4,202, respectively, as confirmed by the expert.

Last, some patients with metal braces or implanted dentures of other materials have artificial materials in their teeth. As shown in Figure 1, we visualized tooth CBCT data by setting different window widths and window levels and then used red and blue boxes to display local image information. In the top row, the window width and window level are set to 3,010 and 2006, respectively. In the bottom row, the window width and window level are set to 3,074 and 4,202, respectively. In the left column, the details of the teeth can be seen in both the red and blue boxes. The red frame image cannot distinguish metal materials in the middle image, while the blue print can better judge the boundary. On the right, the picture in the red box can better represent the teeth, while the tooth in the blue box is partially missing. These interferences may increase the error of tooth segmentation and reduce the effect of tooth segmentation.

To address these challenges, we tried to optimize the local spatial information of dental CBCT images. This study focused on local feature enhancement, given that teeth have significantly different arrangements and indistinguishable boundaries. First, we study some classical local enhancement algorithms. On this basis, a more suitable local enhancement module for tooth segmentation is proposed. Then, the local enhancement module is further integrated into the encoding–decoding framework. At the same time, the metal material between the teeth was considered. By separating metal materials, the convolutional network can automatically learn whether the patient’s tooth section contains metal materials to further improve tooth segmentation.

We summarize the contribution as given below; 1) this study proposes a local contextual enhancement module for clinical tooth CBCT images. We analyzed the distribution of tooth images and further suggested a local enhancement module that is more suitable for dental CBCT images. 2) We verified the existed works and integrated the local enhancement module into the encoding–decoding framework for tooth CBCT images. 3) We validated the superiorities of our method over the state-of-the-art methods through extensive experiments on the built CBCT database.

## 2 Related works

As the foundation of tooth CBCT image analysis, tooth segmentation plays an essential role for dentists in determining orthodontic treatment plans. Traditional tooth image segmentation methods can achieve effective tooth segmentation. However, with the continuous rise of deep learning, its excellent modeling ability and generalization have also received extensive attention. The following subsections will highlight the classical methods and CNN architectures for tooth segmentation.

### 2.1 Literature on classical methods

As early as the 1960s, image segmentation attracted extensive attention in the academic community. Early segmentation methods segmented images between regions according to the features of the image, such as grayscale, color, texture, and shape. Classical methods are differentiated into several categories.

1) The threshold-based segmentation method. The threshold-based method sets the threshold by extracting the grayscale features of the image. The gray value of each image pixel is compared with the threshold and assigned to the corresponding category. Tikhe et al. (2016) selected a single threshold by the pixel gray level of the histogram to identify enamel caries and proximal caries. Mao et al. (2018) designed an iterative segmentation method based on a global threshold. By synthesizing and median-filtering the segmented complete tooth contour image and crown image, the problem of adjacent tooth crown adhesion is solved to a certain extent. However, the aforementioned two methods are limited by a single threshold and cannot effectively separate the tooth and background regions.

In the dental panoramic X-ray image, the contrast between the tooth and background areas is significantly different at the root and crown positions. Therefore, it is necessary to adaptively select different thresholds for different image positions to obtain more accurate segmentation results. From a multi-threshold perspective, Mohamed razali et al. (2014) and Indraswari et al. (2015) used a locally adaptive threshold segmentation method to segment dental panoramic X-ray images, which are used in different segmentation objects and scenes. The final segmentation result error is lower than that of single threshold segmentation.

The threshold segmentation method has a simple model, fast running speed, and without annotation requirements. However, due to the method’s limitations, only simple scenes with a few categories can be segmented, and the processing of more complex scenes with foreground and background is weak.

2) The edge-based segmentation method. This method first detects the apparent differences in grayscale, color, and texture of different types of objects. On this basis, the discontinuous region in the image is detected by pixel gradient differentiation and other methods to find the image’s edge to achieve the target region’s segmentation. Ali et al. (2015) made full use of the active contour model to solve the problem of weak and insignificant gradients between teeth and their backgrounds. Lin et al. (2012) proposed a fully automatic tooth segmentation model with three coupled level set functions. Subsequently, Rad et al. (2013) offered to extract the features of teeth using the grayscale co-occurrence matrix. The aforementioned methods easily fall into local minima and slowly converge. In addition, the most typical edge-based segmentation method is based on the level set method, (Li et al., 2007) which combines the level set and the support vector machine (SVM) to solve the problem subtly. It provides the initial contours for the two coupled level sets, improving tooth segmentation accuracy and time. Such methods also use the underlying features to calculate the edge, which is challenging when dealing with complex scenes.
3) The area-based method. According to the similarity criterion of gray image features, this method looks for the maximum consistency region and then divides the image into different regions. The main methods include region split and merge (Chen and Pavlidis, 1979), seeded region growing (Adams and Bischof, 1994), and watershed algorithm (Meyer and Beucher, 1990). Li et al. (2012) proposed the top-hat–bottom-hat transform to amplify the contrast between the foreground and background, improving the segmentation by removing the noise before a watershed algorithm. Subsequently, Radhiyah et al. (2016) used Gaussian filtering and histogram equalization filtering to preprocess dental panoramic X-ray images. However, the aforementioned methods cannot effectively eliminate the sensitive noise of the watershed algorithm.
4) The clustering-based method. The clustering method transforms the image to be segmented from the image space to the feature space and clustering features in the feature space through similarity, e.g., Euclidean distance and correlation coefficient. Finally, the clustering results are mapped back to the image space to segment the image. Based on the aforementioned principle, segmentation depends on the clustering and similarity measurement methods used. In addition, the construction of the feature space can be one-dimensional or multi-dimensional, and the grayscale, texture, color, depth, and combination of the image can be used as the basis for the construction of the multi-dimensional feature space. Son and Tuan, (2016) used semi-supervised entropy-regularized fuzzy clustering to segment teeth, but this method still requires manual intervention and has certain limitations. Subsequently, Alsmadi (2018) used the fuzzy c-means clustering method to segment the injured jaw in the panoramic X-ray image automatically. Their approach can perform well without blurring the edge of the segmentation target.

### 2.2 Literature on CNN-based methods

Many researchers have recently contributed to tooth segmentation with deep learning methods. Compared with classical tooth segmentation methods that need to set complex rules for modeling, data-driven CNN-based methods have more vital modeling ability and generalization ability. Wirtz et al. (2018) combined a coupled shape model with a neural network, and they combined the features of gradient images with prior statistical knowledge to build a segmentation model. However, this segmentation model cannot segment wisdom teeth. Lee et al. (2020) established a novel method to estimate the average gray density level in the bone and tooth regions. Zhang et al. (2020) developed CNN’s intuitive 3D tooth segmentation approach in harmonic parameter space. They built a 3D tooth model with 2D harmonic parameter space in tooth images and constructed the CNN to study how to perform high-quality and robust tooth segmentation automatically and precisely. Chen et al. (2020) achieved automatic segmentation for the individual tooth in CBCT images by a multi-task 3D fully convolutional network. Rao et al. (2020) proposed a novel symmetric full convolutional network with residual block and dense conditional random field. This method can achieve automatic tooth segmentation because of particular deep bottleneck architectures and summation-based skip connections. Gu et al. (2021) proposed a tooth segmentation method on the dental mesh model. They used an improved region growing algorithm and parameter adaptive method to expand the resemble regions and remove unnecessary parameters to enhance their segmentation performance. To cost-effectively improve the results of tooth segmentation, Li et al. (2021) proposed a group transformer to achieve advanced performance on tooth root segmentation. Koch et al. (2019) input the original image into the network in blocks and achieved pretty performance through U-Net. Although the segmentation result of the tooth is essential for determining the root resorption rate and the localization of the root position, the aforementioned methods still failed to solve the accuracy problem of the fuzzy root. Zhao et al. (2020) used long short-term memory (LSTM) to build an attention mechanism segmentation network that solves the overall low contrast problem in dental panoramic X-ray images. Although this method utilizes short-range feature points to obtain more contextual information, the correlation between distant feature neighborhoods cannot be considered. Cui et al. (2019) first extracted edge maps from input CBCT images to enhance image contrast along shape boundaries. Then, they extracted features from the edge map and input the image separately to learn a new similarity matrix to reduce the number of redundant proposals in the RPN network, speeding up training, and saving GPU memory. Subsequently, Cui et al. (2021b) proposed a two-stage framework including a distance-aware centroid prediction module and a confidence-aware cascade segmentation module to extract all teeth from tooth models with significant variations. The first stage detects all teeth using a distance-aware tooth centroid voting scheme, capable of locating teeth at irregular locations on the abnormal tooth model. Moreover, a confidence-aware cascade segmentation module is designed in the second stage to segment each tooth. Recently, Cui et al. (2022) proposed a multi-level morphology to guide the tooth segmentation model, which characterized the tooth shape from different angles of “point, line, and surface” and accurately extracted the patient’s dental crown and tooth root information.

## 3 The proposed method

The proposed method is a 2D CNN-based framework to segment the tooth and artificial materials from CBCT images. First, the framework is introduced in Section 3.1. Second, the local contextual module is presented in Section 3.2, which enhances the local context information for tooth images through different convolutions. Finally, the loss function of our network is described in Section 3.3, which exploits the pixel potential between various teeth.

### 3.1 Model overview

**FIGURE 2:**
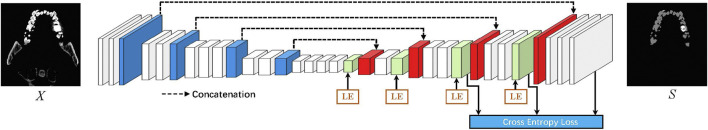
Overview of the proposed tooth segmentation pipeline. LE denotes the local enhancement module. We first designed a local context enhancement module for tooth CBCT images and then integrated the proposed module into the encoder–decoder network. In addition, various decoding modules are optimized through cross-entropy loss.

The critical insight of this study is that local information benefits tooth segmentation, as teeth’ local structure and surface area are closely related. To this end, we design a local context enhancement module to explore the effectiveness of local information on tooth CBCT images. An overview of the proposed method is shown in Figure 2. We assume *X* as the input to the network and *S* as a segmented result for the tooth CBCT slice image. We aim to train a CNN network to segment tooth and artificial metal materials from *X*. We design the LE module to enhance the convolutional local context information on the encoding module. At the same time, the LE module is further embedded into the encoder–decoder network. Finally, we use the cross-entropy loss function in each decoding module to optimize the convolutional features at different resolutions.

**FIGURE 3:**
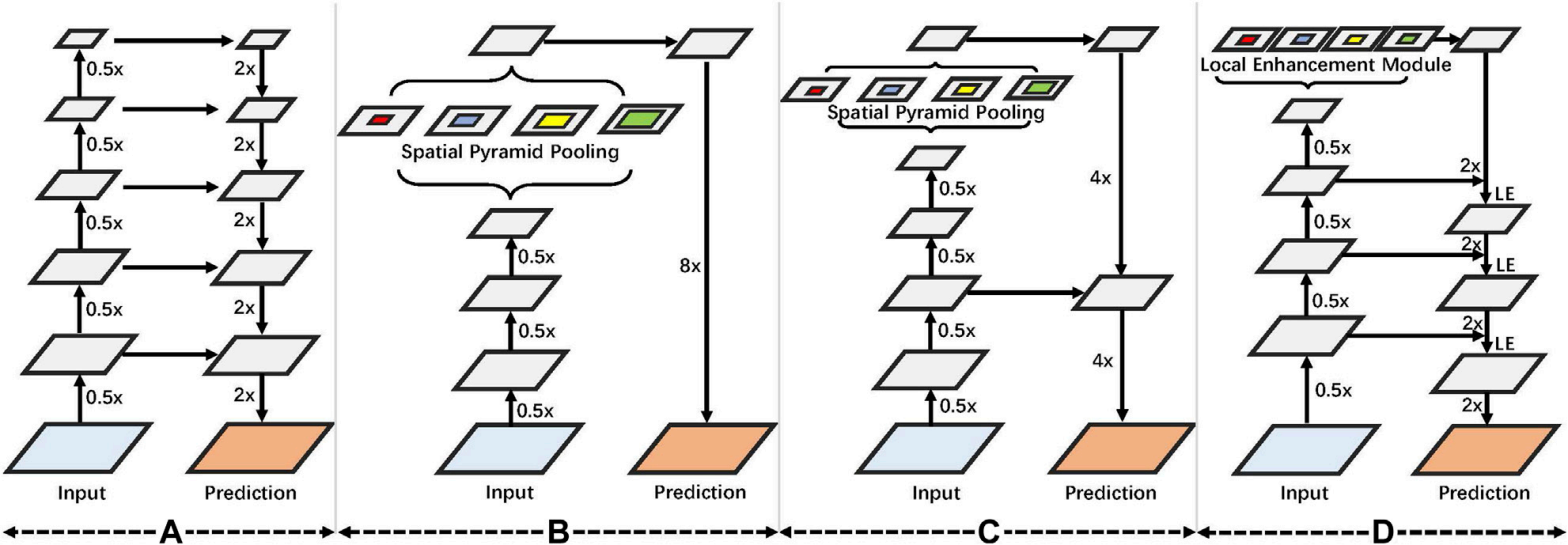
**(A)** We first introduce U-Net, which uses an encoder–decoder framework. **(B)** Used spatial pyramid convolution. **(C)** Coding and decoding structure is further increased based on the spatial pyramid. **(D)** On the proposed local enhancement module, an encoder–decoder design is used.

This study aimed to optimize several existing models, including U-Net (Ronneberger et al., 2015), DeepLabV3 (Chen et al., 2017), and DeepLabV3+ (Chen et al., 2018). As shown in Figure 3A,U-Net uses an encoder–decoder model, which uses feature calculation in the encoder part and then passes through a U-shaped network to gradually restore the clear object boundaries in the decoder part. Figure 3B, *i.e.,* DeepLabV3 proposed the atrous spatial pyramid pooling (ASPP) method, which uses several atrous convolutions on low-resolution features to capture contextual information at multiple scales, considering the constraints of existing GPU memory. Then, the image’s resolution is restored by 8x upsampling, which effectively reduces the computational complexity of the model. However, directly performing 8x upsampling will also lead to poor boundaries of the predicted image, and the model accuracy will also be affected. Based on the work of DeepLabV3, DeepLabV3+ adds a simple, yet effective decoder module to recover object boundaries shown in Figure 3C. While improving the model’s accuracy, it also considers the model parameters of the network. In this study, a feature local enhancement module is designed based on fully considering the structural information of tooth CBCT. Compared with ASPP, the proposed method pays more attention to enhancing the local information of features shown in Figure 3D. First, suppose a dilated convolution with a significant dilation rate is used for low-resolution features, then the spatial correlation between each local pixel and local pixels cannot be fully considered. Second, compared with the farther pixels, the closer pixels can better-correlate the boundary information of the teeth. At the same time, tooth segmentation tasks pay more attention to the model’s accuracy. To this end, we refer to U-Net, which gradually recovers the spatial resolution of the tooth segmentation network in the decoder part. Particularly, we modify the ASPP module based on DeepLabV3+. The improved module focuses more on the enhancement of local features. Meanwhile, the local enhancement module is embedded in the decoder network, and the boundary information of the teeth is gradually recovered.

### 3.2 Local enhancement module

The component of our method is the local enhancement (LE) module to enhance the contextual information of the intermediate feature *X*_*t*_ and output the next module’s feature *X*_*t*+1_.

**FIGURE 4:**
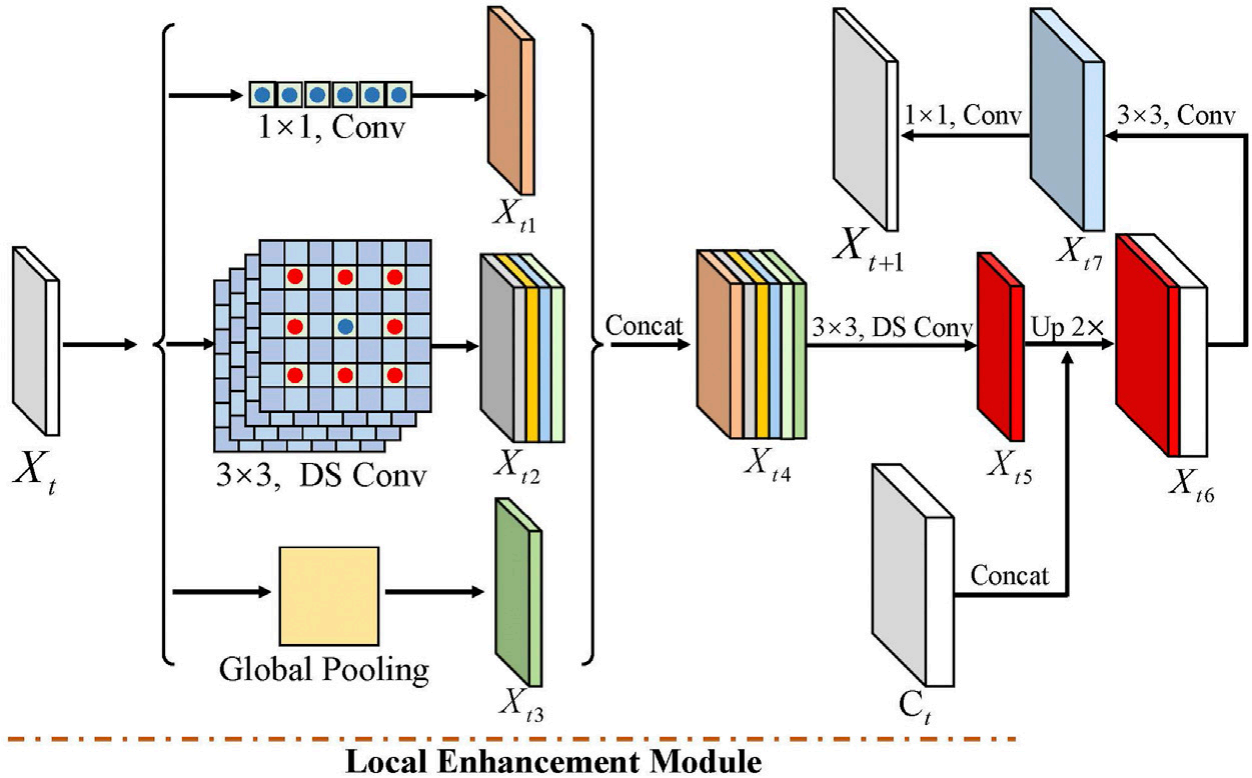
Local enhancement module contains three part convolutions, including 1 × 1, four 3 × 3 depthwise separable convolutions with dilated rates 1, 6, 12, and 18, and global pooling. Among them, DS-Conv denotes depth-separable convolution.

LE enhances the local contextual information from each encoder feature *X*_*t*_. More specifically, LE consists of a 1 × 1 convolution, four 3 × 3 depthwise separable convolutions with dilated rates 1, 6, 12, and 18, and a global pooling. As shown in Figure 4, 1 × 1 convolution changes the channel’s number. 3 × 3 depthwise separable convolution with the dilated rate 1 performs convolution with eight adjacent points. In contrast, other 3 × 3 depthwise separable convolutions conduct convolution operation with the adjoining point of larger intervals, i.e., 6, 12, and 18, which enhances the local relation of tooth features in various resolutions. Compared to 3 × 3 depthwise separable convolution with the dilated rate 1, other 3 × 3 depthwise separable convolution costs similar computations with the same input and output channels. Moreover, we extract the larger receptive field of intermediate features by global pooling.

Among them, *X*_*t*_ is the input in the *t*th block, and *X*_*t*1_, *X*_*t*2_, and *X*_*t*3_ are the output features of 1 × 1 convolution, four 3 × 3 depthwise separable convolutions, and global pooling, respectively. To further reduce the computation cost, we decrease the output channel of *X*_*t*1_, *X*_*t*2_, and *X*_*t*3_. In detail, these features with the same channel can be concatenated to feature *X*_*t*4_. Then, we adopt a 3 × 3 depthwise separable convolution to output the feature *X*_*t*5_, which reduces the channel to one-sixth of *X*_*t*4_. This operation also enhances the local information of the LE module. Next, we integrated the LE module into the encoder–decoder network to gradually restore the tooth feature’s resolution. We first upsampled the feature *X*_*t*5_ by a factor of 2 and then cascaded it with the middle feature *C*_*t*_ of the encoder module to obtain the feature *X*_*t*6_, while the *C*_*t*_’s resolution is twice that of *X*_*t*5_. To further optimize the local features, we used a 3 × 3 convolution to output feature *X*_*t*7_. Finally, the next modules’ feature *X*_*t*+1_ is obtained through a 1 × 1 convolution. In the last three convolutions of the decoder module, the BN layer and the RelU activation function are used.

### 3.3 Loss function

We treat the tooth segmentation problem as a process to distinguish pixel categories. We apply Softmax cross-entropy loss to learn robust features to transfer the semantic knowledge from the ground truth to the network at the pixel level. The pixel-wise loss is defined as

$$L_{pi}=-\frac{1}{HW}\overset{H}{\underset{i=1}{\sum }}\overset{W}{\underset{j=1}{\sum }}\overset{K}{\underset{k=1}{\sum }}\frac{e^{q_{i,j}^{k}}}{\sum e^{q_{i,j}^{k}}}\log\frac{e^{p_{i,j}^{k}}}{\sum e^{p_{i,j}^{k}}},$$ (1)

where *H* and *W* denote the ground truth’s height and weight and the predicted map, respectively, and *K* is the number of the predicted categories. *i* and *j* denote the matrix’s *i*th row and *j*th column, respectively. *p* denotes the predicted map, and *q* denotes the ground truth. $p_{i,j}^{k}$ and $q_{i,j}^{k}$ are the probability values in the *k*th channel of *p* and *q*, respectively.

Following the previous work, auxiliary losses in shallow layers could optimize the learning process, while the main branch loss takes the most responsibility. Therefore, except for the main branch, we apply softmax cross-entropy loss to train the final classifier. We also add weight to balance another auxiliary loss after 3 × 3 convolutions. We set *p*_*t*_ and *q*_*t*_ as the predicted map and ground truth in the *t*th classifier, respectively. The pixel-wise loss in the *t*th classifier is defined as

$$L_{pi}^{t}=-\frac{1}{H_{t}W_{t}}\overset{H_{t}}{\underset{i=1}{\sum }}\overset{W_{t}}{\underset{j=1}{\sum }}\overset{K}{\underset{k=1}{\sum }}\frac{e^{q_{i,j}^{t,k}}}{\sum e^{q_{i,j}^{t,k}}}\log\frac{e^{p_{i,j}^{t,k}}}{\sum e^{p_{i,j}^{t,k}}},$$ (2)

where *H*_*t*_ and *W*_*t*_ denote the height and weight of *p*_*t*_ and *q*_*t*_ in the *t*th classifier, respectively. $p_{i,j}^{t,k}$ and $q_{i,j}^{t,k}$ are the probability values in the *k*th channel and *t*th classifier of *p*_*t*_ and *q*_*t*_, respectively. It should be noted that *q*_*t*_ is directly downsampled by bilinear interpolation from the original ground truth *q*, and *q*_*t*_ is the same resolution with the current prediction *p*_*t*_. The total pixel-wise loss is defined as

$$L_{pi}^{T}=\overset{\tau }{\underset{t=1}{\sum }}\mu_{t}L_{pi}^{t},$$ (3)

where $L_{pi}^{T}$ is the total loss function in the pixel-wise level. *μ*_*t*_ is the weight of $L_{pi}^{t}$ , 1 ≤ *t* ≤ *τ* − 1, which gradually increases as *t* becomes larger. We set *μ*_*t*_ to 0.2 and 0.3 during training. We abandoned those auxiliary branches in the testing stage and only used the last branch as the final prediction.

## 4 Experiments

We conducted experiments on a clinical dental CBCT dataset collected in a hospital, and at the same time, we benchmarked the segmentation results with several state-of-the-art methods. We also evaluated the impact of the proposed method on our model. Finally, we discussed the following works and future research directions in the discussion.

### 4.1 Data description

Currently, there are few publicly available tooth segmentation datasets with pixel-level markings. To this end, we constructed a new tooth CBCT segmentation dataset. In this section, we introduced data collection and professional marking.

A. Data collection: to protect patients’ privacy, we ignored their personal information in the dataset. We collected CBCT images of 100 patients. All data were acquired at a Chinese hospital between January and November 2021. A medium sharp reconstruction algorithm reconstructed the CBCT slices with a thickness of 0.3 mm.
B. Data annotation: although we have captured enough tooth CBCT data, accurately annotated labels are essential for deep learning. To this end, we formed a team of two annotators with deep radiological backgrounds and proficient annotation skills to annotate the areas and boundaries of the tooth CBCT image. A senior radiologist checked the final annotation with first-line clinical experience. For the segmentation task, we performed pixel-level labeling as strategies: 1. to save labeling time, the radiologist randomly selected CBCT scan images of 11 patients. In this step, our goal is to label the infected areas with pixel-level annotations. 2. To generate high-quality annotations, we invited a senior radiologist to refine the labeling marks for cross-validation. Some inaccurately labeled images have been removed in this stage.

After the aforementioned annotation procedures, we finally obtained 3, 024 pixel-level labeled CBCT slices from 11 patients with a resolution of 410 × 410. Among them, only 1, 660 slices contain a tooth. We only selected images with tooth labeling for training and testing to reduce the labeling process’s errors. We randomly split the dataset into nine patients for training and two patients for testing. Among them are 1, 360 training images and 300 images for the test.

### 4.2 Evaluation metrics

1) Intersection and union ratio: it is one of the most commonly used indicators for semantic segmentation. It calculates the ratio of the intersection and union of pixel sets between the prediction space and the labeled space. The IoU of the *i* category is defined as follows:

$$IoU_{i}=\frac{p_{i,i}}{\sum_{j=0}^{k}p_{j,i}+\sum_{j=0}^{k}p_{i,j}-p_{i,i}}.$$ (4)

2) Average cross-union ratio: the average cross-union ratio is calculated from the cross-union ratio. First, the IoU value of each category is calculated and then the IoU value of each category is averaged to calculate mIoU. The formula is calculated as follows:

$$mIoU=\frac{1}{k}\overset{k-1}{\underset{i=0}{\sum }}\frac{p_{i,i}}{\sum_{i=0}^{k}p_{j,i}+\sum_{j=0}^{k}p_{i,j}-p_{i,i}}.$$ (5)

### 4.3 Training detail

Our model is implemented with TensorFlow-GPU 2.4.0. All training and testing are carried out on a single TITAN RTX GPU using CUDA 11.0 and CUDNN 8.0. In detail, we train the network parameters over 500 epochs using the training set with a descending learning rate. The initial value of the learning rate is equal to 0.001. We utilized the stochastic gradient descent (SGD) with a momentum of 0.99 and a weight decay of 0.0005 in training for all experiments. The batch size is set to 8.

### 4.4 Comparison to state-of-the-art methods

We carried out experiments on the tooth dataset with other state-of-the-art methods to show the proposed approach’s effectiveness. As shown in Table 1, we compared our result with other state-of-the-art methods, i.e., U-Net (Ronneberger et al., 2015), DenseASPP (Yang et al., 2018), BiSeNet (Yu et al., 2018), PSPNet (Zhao et al., 2017), PAN (Li et al., 2018), DeepLabV3 (Chen et al., 2017), DeepLabV3+ (Chen et al., 2018), and UNeXt (Valanarasu and Patel, 2022). These codes are available online, and we follow the authors’ instructions to train the models on the tooth CBCT dataset. It should be noted that we evaluate tooth segmentation accuracy with three famous metrics in medical imaging analysis, including IoU and mIoU.

**TABLE 1:** Segmentation comparison among our method and other state-of-the-art methods on the COVID-19 dataset.

Method	IoU_*back*_	IoU_*tooth*_	IoU_*metal*_	mIoU
U-Net Ronneberger et al. (2015)	99.35	80.89	58.91	79.68
DenseASPP Yang et al. (2018)	98.76	64.88	45.48	69.71
BiSeNet Yu et al. (2018)	98.63	62.43	24.44	61.83
PSPNet Zhao et al. (2017)	98.95	69.37	42.44	70.26
PAN Li et al. (2018)	99.26	77.24	52.32	77.27
DeepLabV3 Chen et al. (2017)	98.20	45.25	21.18	54.88
DeepLabV3 + Chen et al. (2018)	99.38	82.12	63.87	81.79
UNeXt Valanarasu and Patel (2022)	99.27	81.56	61.34	80.72
Ours	**99.51**	**86.37**	**68.34**	**84.74**

Bold values indicates the best performing parameter.

Among those compared methods, U-Net, UNeXt, and DeepLabv3+ gain a more competitive result than others. U-Net and UNeXt gradually recover the boundary information of the image through the encoder–decoder architecture. DeepLabv3+ uses ASPP to enhance the contextual information and adopts the decoder module to optimize the network. Moreover, DeepLabv3 also uses ASPP but does not use the decoding module, significantly affecting accuracy. Similarly, DenseASPP, PSPNet, and PAN also use the spatial pyramid module but do not fully consider the design of the decoding module, resulting in poor performance of tooth segmentation. However, BiSeNet has designed lightweight network architecture, resulting in poor feature extraction capabilities of the network. For tooth segmentation, more attention is paid to accuracy, which can provide reasonable assistance for clinicians in diagnosis and treatment. To this end, this study further designs a local enhancement module based on ASPP. At the same time, referring to the structure of U-Net, the boundary information of the image is restored step by step.

**FIGURE 5:**
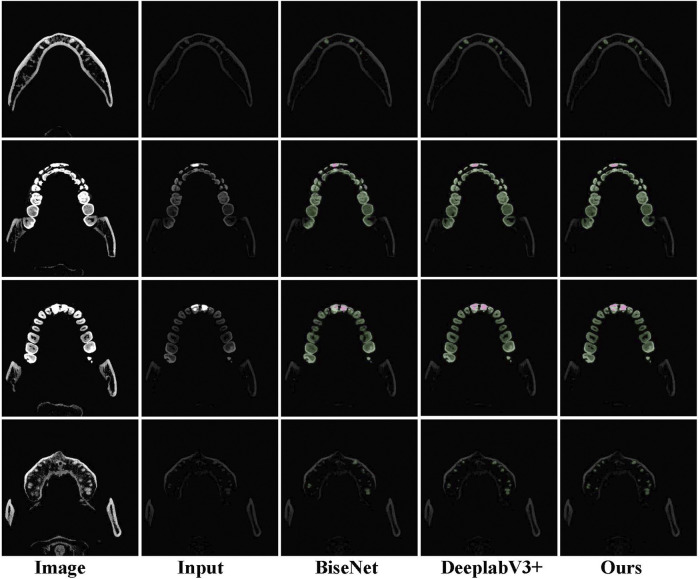
Examples of tooth segmentation are predicted by our method with other state-of-the-art approaches.

In terms of IoU and mIoU, our network achieves the best performance, demonstrating the superiority of the method in tooth segmentation. Moreover, some visual comparisons among U-Net Ronneberger et al. (2015), BiSeNet Yu et al. (2018), DeepLabV3+ Chen et al. (2018), and our method are displayed in Figure 5. The first column is used for reference by setting different window widths and window levels. The second column serves as the input to the network. It can be seen that the accuracy of our method and DeepLabv3+ is significantly better than that of BiSeNet.

### 4.5 Ablation study

In this section, we first analyze the effects of different components in the proposed method. To examine the local enhancement (LE), we report the experimental results in Table 2. For the base network, the U-Net-based framework is adopted for our network, and the Softmax cross-entropy loss is merely used for training. We use ResNet151 as the encoder network and one decoder module on the decoder network. The decoder module has two convolutional layers, i.e., a 3 × 3 deconvolution with stride two and a 3 × 3 convolution with stride 1. It should be noted that a 3 × 3 deconvolution is used to restore the resolution of the feature. LE modules with an encoder–decoder network significantly contribute to the excellent performance. Among them, Base + LE only computes in the low-resolution feature, while “Base + LE (decoder)” applies an LE module in different decoder stages, which proves the various local enhancement of the whole network. From Table 2, LE obtains 0.17%, 3.85%, and 5.62% improvement in IoU_*back*_, IoU_*tooth*_, and IoU_*metal*_, respectively, when LE is applied in decoder modules and further gets 0.12%, 3.65%, and 4.41%, respectively, improvement when LE is adopted in both decoder modules.

**TABLE 2:** Ablation study of our method. “Base” is trained with only the Softmax cross-entropy loss, and “Base + LE” denotes the base network using a local enhancement module. “Base + LE (decoder)” represents that the LE is embedded into the encoder–encoder network.

Method	IoU_*back*_	IoU_*tooth*_	IoU_*metal*_	mIoU
Base	99.21	78.87	58.31	78.79
Base + LE	99.39	82.72	63.93	82.01
Base + LE (decoder)	**99.51**	**86.37**	**68.34**	**84.74**

Bold values indicates the best performing parameter.

Then, we explored the loss function’s influence on the experimental results, including IoU loss and cross-entropy loss. As shown in Table 3, IoU loss improves by 82.45% and 86.15% in IoU_*tooth*_ and IoU_*metal*_, respectively, while the cross-entropy loss makes a 3.7% and 6.99% improvement, respectively. Moreover, the tooth segmentation network achieves the best result when the auxiliary loss is applied. IoU loss cannot fully explore the supervision information of labeled data, and its accuracy is lower than that of cross-entropy loss. At the same time, the auxiliary loss can optimize features at multiple resolutions, improving the network’s accuracy to a certain extent.

**TABLE 3:** Ablation study of various losses. Cross-entropy loss (auxiliary) denotes our network using cross-entropy loss with two auxiliary losses.

Method	IoU_*back*_	IoU_*tooth*_	IoU_*metal*_	mIoU
IoU loss	99.35	82.45	61.25	81.02
Cross-entropy loss	99.42	86.15	68.24	84.60
Cross-entropy loss (auxiliary)	**99.51**	**86.37**	**68.34**	**84.74**

Bold values indicates the best performing parameter.

Finally, we tried to test the trained model on two different ground-truth data. Ground-truth 1 represents the data marked with the first column image in Figure 5, and ground-truth 2 uses the second column data for marking. The experimental results are shown in Table 4. After testing with ground-truth 2 data, tooth segmentation accuracy significantly improved because the data in the first column can better show the outline of the teeth but cannot exhibit the information on metal fillings. On the contrary, the second column data can better-highlight the metal fillings after adjusting the window width and window level, but missing in the tooth contour. Therefore, we used ground-truth 1 annotations in the previous experiments for training and testing.

**TABLE 4:** Our method was tested on different ground-truth.

Method	IoU_*back*_	IoU_*tooth*_	IoU_*metal*_	mIoU
Ours (ground-truth 1)	99.51	86.37	68.34	84.74
Ours (ground-truth 2)	**99.58**	**92.34**	**69.12**	**87.01**

Bold values indicates the best performing parameter.

## 5 Discussion

Automatic segmentation of medical images has attracted numerous researchers in recent decades, assisting doctors or patients in understanding the medical data. We presented an encoder–decoder framework based on local feature enhancement in this work. This network aimed to fully devote the accurate semantic and location contexture information over the input image.

Then, we used the auxiliary loss function to optimize the semantic spatial information in different resolutions. In this way, we obtained few improvements in tooth segmentation. We used a local enhancement module to refine the tooth contexture information and the edge step by step. From the quantitative comparison between the proposed method and the other three latest methods, we can conclude that the proposed method is superior to other methods. The main reason is that our network can optimize the receptive field to various resolutions.

However, limited by the scale of the tooth segmentation data, the proposed algorithm still has room for improvement. In addition, the annotation of tooth segmentation data can be improved. As shown in Figure 5, we apply the data in the first column to label the tooth image, and the second column images are used to mark the filling metal material, which leads to boundary errors when predicting the tooth segmentation from the second column images. Moreover, the proposed method is based on the two-dimensional tooth segmentation network, which fails to consider the information changes of the tooth segmentation in the three-dimensional space. In the future, we will continue to study the instance segmentation of oral CBCT images based on the research of this study. At the same time, we will explore how to construct 2D convolutional networks to learn the spatial variation between different slices.

## 6 Conclusion

We have proposed a practical local enhancement module for tooth segmentation, which explores the local relationship of the different teeth. Based on ASPP, LE further considers the local correlation between different teeth. At the same time, the proposed network uses an encoder–decoder module to recover the boundary information of the tooth image level by level. The experimental results have demonstrated the superior performance of our method.

## Data Availability

The raw data supporting the conclusion of this article will be made available by the authors, without undue reservation.
